# Will a large complex model ecosystem be viable? The essential role of positive interactions

**DOI:** 10.1002/ecy.70064

**Published:** 2025-03-19

**Authors:** Rudolf P. Rohr, Louis‐Félix Bersier, Roger Arditi

**Affiliations:** ^1^ Department of Biology University of Fribourg Fribourg Switzerland; ^2^ Institute of Ecology and Environmental Sciences (iEES‐Paris), Sorbonne Université Paris France

**Keywords:** complexity, model ecological communities, mutualistic interactions, persistence, positive interactions, prey–predator interactions, viability

## Abstract

Ecologists have documented many characteristics of natural systems that foster ecosystem persistence, and it might be deduced that such strategies are essential for counteracting the negative effect of complexity on local stability that was suggested by R.M. May in his influential work of the 1970s. However, we show that the loss of local stability does not necessarily imply total ecosystem extinction. A more general criterion of ecosystem viability is the long‐term persistence of any number of surviving species—not necessarily all of them. With this approach, we show that persistence increases with complexity, contrary to previous theoretical findings. In particular, positive interactions (mutualistic or prey‐to‐predator) play a crucial role in creating ecological niches, which sustain biodiversity with increasing complexity.

## INTRODUCTION

The relationship between complexity and stability has long been debated in ecology (Allesina & Tang, 2012; Cohen & Newman, 1985; Elton, 1958; Hatton et al., 2024; MacArthur, 1955; May, 1972, 1973; McCann, 2000; Mougi & Kondoh, 2012, 2014; Odum, 1953; Pimm, 1984; Tilman & Downing, 1994; Tregonning & Roberts, 1979). Laboratory and field experiments (Hooper & Vitousek, 1997; McCann, 2000; McGrady‐Steed et al., 1997; Naeem et al., 1994, 1995, 1996; Naeem & Li, 1997; Tilman et al., 1996) tend to indicate that more diverse ecological communities are more productive and less variable. These findings are in line with the conventional wisdom of ecologists that greater biodiversity favors ecosystem health (Elton, 1958; MacArthur, 1955; Odum, 1953). However, these findings also seem to contradict the pioneering theoretical studies (Gardner & Ashby, 1970; May, 1972, 1973) that demonstrated that randomly built complex mathematical models tend to be unstable and, thus, that the corresponding ecosystems are unlikely to exist in nature. The rationale originally used by May (1972) to prove the nonviability of large complex systems was as follows: take *S* dynamic variables with random interactions, assume that they are in equilibrium, and examine the local (mathematical) stability of this equilibrium. Pairwise interactions can be of all types: competition (− −), antagonism (− +), mutualism (+ +), commensalism (+ 0), and amensalism (− 0). An equilibrium is locally stable if, after a small disturbance, the system returns to it. The authors who used this approach (Gardner & Ashby, 1970; May, 1972, 1973) observed that the probability of being in a stable local equilibrium decreases when the number of species *S* increases and/or the connectance *c* (a measure of the interaction density) increases.

Most importantly, these authors did not investigate the nonequilibrium properties of unstable systems because they did not follow the time course of population trajectories. The limited power of computers at that time only permitted determining whether equilibria were stable or unstable. Resting on the assumption that existing ecological systems in nature are necessarily stable and equating viability with local stability, the conclusion was that complexity could not be an explanation for ecological viability.

Here, we argue that it is incorrect to dismiss all unstable systems: some unstable systems can be viable. Taking the case of a single population as the simplest possible example, the basic model of population biology is the Malthus equation.

$$\frac{\mathrm{dN}}{\mathrm{Ndt}}=\lambda ,$$

which predicts either unlimited growth (if λ>0) or a decline to extinction (if λ<0) but never nonzero stable equilibrium. With the help of a model, every conservation ecologist would be happy to find that some endangered species has a growth rate λ>0, suggesting that the species is not on the way to extinction. However, this also means, strictly speaking, that the population is on the way to infinity and, thus, is unstable. In this case, instability does not equate with extinction, on the contrary! Of course, the population must ultimately encounter some limitation to growth, which is not accounted for in the basic Malthus equation. Simply put, every model has a realm of applicability and, in a conservation context, when populations are low, the Malthus equation is sufficient.

How can this argument for a single population be extended to a multispecies community? Will an unstable system result in total extinction, or will some species grow indefinitely and thus be viable? If the latter is true, how many species are involved in the collective growth? What are the factors that determine the probability of this growth? What happens when growing populations ultimately encounter unavoidable limitations?

In this paper, we argue that many of the theoretical systems that were considered nonviable because of their instability actually correspond to biologically viable systems: those systems that “escape” from the local (mathematically unstable) equilibrium, with one or more species growing indefinitely. Of course, exploding species must ultimately reach bounds set by limiting resources that were not accounted for by the theoretical models. The only nonviable systems are those in which *all* populations decline to extinction.

## METHODS

A first building block in the mathematical description of a multispecies situation is the well‐known Lotka–Volterra family of models for two species. Depending on the signs of the parameters that quantify the interspecific interactions, these models can describe all pairwise interactions mentioned in the Introduction (Gause & Witt, 1935; May, 1973, 1981; Odum, 1953). The dynamic outcomes of these systems are well known. Antagonism (e.g., predation) can lead to stable coexistence, cycling coexistence, or extinction. Situations with negative interactions only (competition, amensalism) lead either to coexistence or to alternative stable states with extinctions (Gause & Witt, 1935). Particularly interesting are the situations with positive interactions only (mutualism, commensalism): these interactions can lead to coexistence, to extinction, or to population explosion (Gause & Witt, 1935; Goh, 1979).

In order to describe the dynamics of larger communities with more than two species, we will use the classical extension known as the generalized Lotka–Volterra model (GLV; see below). Note that we assume that any pair of species is linked by a single type of interaction (competition, antagonism, mutualism, …). We do not consider dual interactions, as defined by Mougi (2024).

We start by giving a general outline of the methodological rationale. First, given a number of species *S*, we sample a per capita interaction strength matrix **A** (of size S×S) and a vector of equilibrium positive abundances N* (of size *S*). Based on this interaction matrix and this equilibrium vector, a unique GLV model is built in such way that N* is a positive equilibrium of this model. Then, the eigenvalues of the Jacobian matrix evaluated at this point are calculated. Given these eigenvalues, the local stability of the equilibrium point is determined by applying the Routh–Hurwitz criterion.

Second, we generate at random a perturbed vector **N**_**p**_ that is very close to the equilibrium vector N*. This perturbed vector is used as the initial condition for simulating numerically the GLV model parameterized as above. If the equilibrium point N* is locally stable, the dynamics converge back, of course, to **N**;* but, if it is unstable, two possibilities can occur:

1. The dynamics converge to another equilibrium point, possibly with some species becoming extinct, or even all of them (system extinction).
2. One or more species can blow up with no limit. In this case, we stop the simulation when any species reaches an abundance of 1000 (chosen as an arbitrarily large number).

At the end of the simulation, each species with an abundance greater than 10^−4^ is considered alive, while if its abundance is lower, it is considered to be extinct.

Third, we repeat these two steps for a gradient of initial community richness *S* ranging from 10 to 250 (or 350) species in increments of 10 and simulate 1000 random communities for each richness *S*.

In order to avoid unrealistic abundance growth to infinity, which can occur in the second step above, we also devised a modification of the GLV model in such a way that populations cannot grow uncontrolled. The assumption is that any species must ultimately encounter limits to growth. In this modified model, each species is bounded to an abundance *N*_max_, with its per capita growth rate declining asymptotically to zero when approaching *N*_max_. This is not mechanistic modeling but the simplest phenomenological model that avoids unlimited growth. The dynamics of this modified model always converge to an equilibrium point. Note that the modified model is built in such a way that the sampled equilibrium point N* at the first step is still an equilibrium point of the modified model.

At the end of the simulation, we count the number of species still alive as well as the number of species that reached the maximum abundance *N*_max_. In our numerical simulations, we set $N_{\max}=1000$. We repeat the simulations as in the third step.

Finally, in order to understand the effects of positive and negative interactions, we repeat the modified GLV model simulations: first, with interaction matrices containing only negative or null interactions, and second, with only positive or null interspecific interactions and negative intraspecific interactions (intraspecific competition).

### GLV model

The dynamic equations of the GLV model are

$$\frac{dN_{i}}{\mathrm{dt}}=N_{i}\times \left(r_{i}+\sum_{j=1}^{S}a_{\mathrm{ij}}\times N_{j}\right).$$

Given a sampled vector N* and a sampled interaction matrix **A**, N* must be a positive equilibrium point of the GLV model. This is ensured by setting the vector of intrinsic growth rates **r** as

$$r=–{\text{AN}}^{*}.$$

Hofbauer and Sigmund (1998, p. 4, Theorem 5.2.1) proved mathematically that, in the GLV model, the existence of a strictly positive trajectory requires the existence of a strictly positive equilibrium point (possibly unstable). In other terms, no trajectory can exist in the absence of an equilibrium. Thus, the existence of an equilibrium is not a restrictive assumption.

The model dynamics are simulated using the numerical integrator lsodsa of the library deSolve (Soetaert et al., 2010) with the R software (R Core Team, 2024). The R code that we used for performing the simulations and for drawing the figures is publicly available (Rohr, 2025). The simulation is stopped when any species reaches an abundance of 1000 or when all species have converged to an equilibrium (i.e., $\left|dN_{i}/\mathrm{dt}\right|<10^{-5},\forall i$). In theory, it is also possible that one or more species settle on a never‐ending cyclic (or chaotic) regime. For this reason, we set a simulation time limit of 1000 steps. However, this situation never occurred in our simulations (see *Discussion*).

### Sampling the interaction matrices and the equilibrium abundances

The per capita interaction strength matrix $A=\left[a_{\mathrm{ij}}\right]$ is generated as follows:

1. We set the diagonal elements, that is, the intraspecific competition strength, to −1; that is, *a*_*ii*_ = −1.
2. The matrix **A** contains *c S* (*S*–1) nonzero off‐diagonal elements *a*_*ij*_. The parameter *c* is the connectance, that is, the proportion of nonzero interspecific interaction coefficients. The magnitude of these interaction coefficients is sampled at random in a Gaussian distribution of mean zero and of variance σ, that is, $a_{\mathrm{ij}}\sim N\left(0,\sigma \right)$.
3. The remaining interspecific interactions are set to zero.

The elements of the equilibrium abundance vector N* = [*N*_i_*] are sampled following a log‐Gaussian distribution with a mean of zero and a SD of 1, that is, $\ln\left(N_{i}^{*}\right)∽N\left(0,1\right)$. Consequently, the equilibrium abundances are, on average, equal to e^1/2^.

### Sampling the perturbations

The initial conditions for simulating the GLV model are set by a perturbation of the equilibrium abundances N*. The elements *N*_*p,i*_ of the vector of perturbed abundances **N**_**p**_ are obtained by adding a normally distributed random number of mean zero and SD 0.02 to the equilibrium abundances *N*_i_*, that is, $N_{p,i}∽N\left(N_{i}^{*},0.02\right)$. Note that, in order to avoid negative abundances in the very rare case in which the sampled perturbed elements are negative (i.e., *N*_*p,i*_ < 0), we take their absolute values.

### Modified GLV

In order to avoid unlimited growth, we use the following modification of the GLV model:

$$\frac{dN_{i}}{\mathrm{dt}}=N_{i}\times \left(r_{i}+\sum_{j}a_{\mathrm{ij}}\times N_{j}\right)\times \left(\frac{N_{\max}-N_{i}}{N_{\max}}\right).$$

Compared with the original GLV model, we apply the multiplicative term $\left(\frac{N_{\max}-N_{i}}{N_{\max}}\right)$. This term avoids unlimited growth by bounding, from above, the abundance of each species to the value $N_{\max}$. When the abundance is below this threshold, the sign of the whole expression is given by the sign of the first bracket, that is, the original GLV model. Moreover, an equilibrium point N* of the original GLV model remains an equilibrium point of the modified model. When a population converges to the abundance $N_{\max}$, its per capita growth rate declines and converges to zero. Thus, this multiplicative term sets the upper bound $N_{\max}$ to every species abundance. In the numerical simulations, we set $N_{\max}=1000$. With no loss of generality, we can assume that all species are renormalized in such a way that they all have the same $N_{\max}$.

**FIGURE 1:**
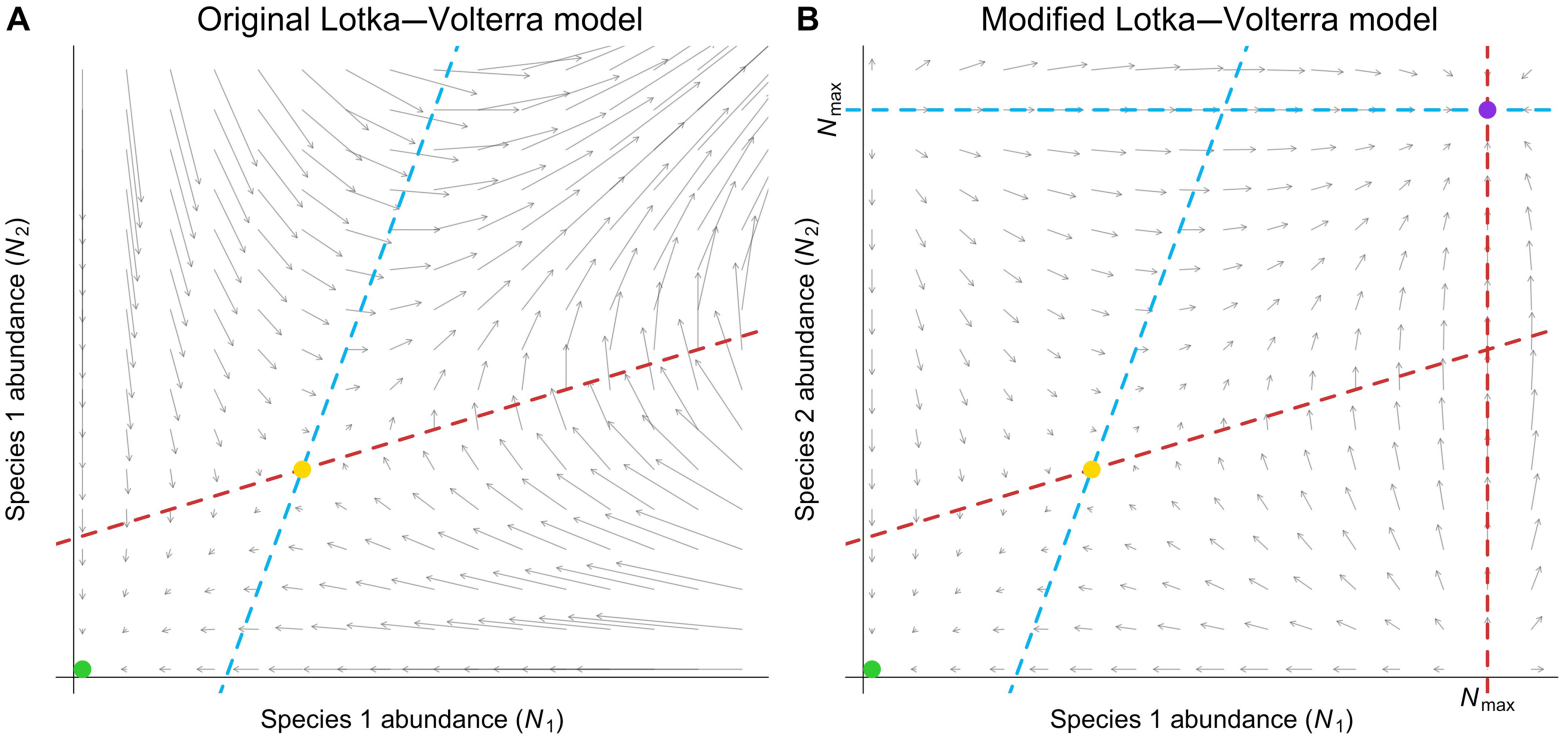
Phase‐plane comparison between the original and the modified Lotka–Volterra models for two mutualistic species. Panel (A) shows, in the original Lotka–Volterra model, the typical pattern with an unstable nontrivial equilibrium point (yellow dot) leading either to population blow‐up at the upper right or toward extinction (green dot). Panel (B) shows the changes brought about by the modified version of the Lotka–Volterra model. The panel clearly shows that the populations are now bounded by the new stable equilibrium point at the upper right (purple dot). Detailed explanations are given in the *Methods* section.

In order to further understand the properties of the modified model, we now perform the phase‐plane analysis of a system of two mutualistic species (Figure 1). The original Lotka–Volterra model is given by the following system of two differential equations:

$$\frac{dN_{1}}{\mathrm{dt}}=N_{1}\times \left(r_{1}+a_{11}\times N_{1}+a_{12}\times N_{2}\right),$$

$$\frac{dN_{2}}{\mathrm{dt}}=N_{2}\times \left(r_{2}+a_{21}\times N_{1}+a_{22}\times N_{2}\right),$$

while the corresponding modified Lotka–Volterra model is

$$\frac{dN_{1}}{\mathrm{dt}}=N_{1}\times \left(r_{1}+a_{11}\times N_{1}+a_{12}\times N_{2}\right)\times \left(\frac{N_{\max}-N_{1}}{N_{\max}}\right),$$

$$\frac{dN_{2}}{\mathrm{dt}}=N_{2}\times \left(r_{2}+a_{21}\times N_{1}+a_{22}\times N_{2}\right)\times \left(\frac{N_{\max}-N_{2}}{N_{\max}}\right).$$

In panels A and B of Figure 1, the blue and red dashed lines represent the nontrivial zero‐growth isoclines for species 1 and 2, respectively. We assume the existence of a positive equilibrium, visualized by the yellow dot at the intersection of the isoclines, and we also assume that this equilibrium is unstable (a saddle point), that is, we assume $a_{12}\times a_{21}>a_{11}\times a_{22}$. The green dot is the trivial equilibrium (abundances equal to zero), which, in this case, is always locally stable. The arrows represent the vector field of the differential equations. In panel A, we see that, depending on the perturbation of the positive equilibrium (yellow dot), the system can either converge to extinction (green dot) or blow up to infinity. Panel B shows the effect of the extra term $\left(\frac{N_{\max}-N_{i}}{N_{\max}}\right)$ on the behavior of the modified model. First, there are two new nontrivial zero‐growth isoclines (blue horizontal and red vertical dashed lines) and their intersection defines a new equilibrium point represented by the purple dot. Second, examining the modification of the vector field, we can see that the system is prevented from blowing up, with the maximum abundance that can be reached being $N_{\max}$. Third, depending on the direction of the perturbation of the unstable equilibrium (yellow dot), the system either converges to the trivial equilibrium (green dot), where both species become extinct, or to the new positive equilibrium (purple dot), where both species are alive.

## RESULTS

**FIGURE 2:**
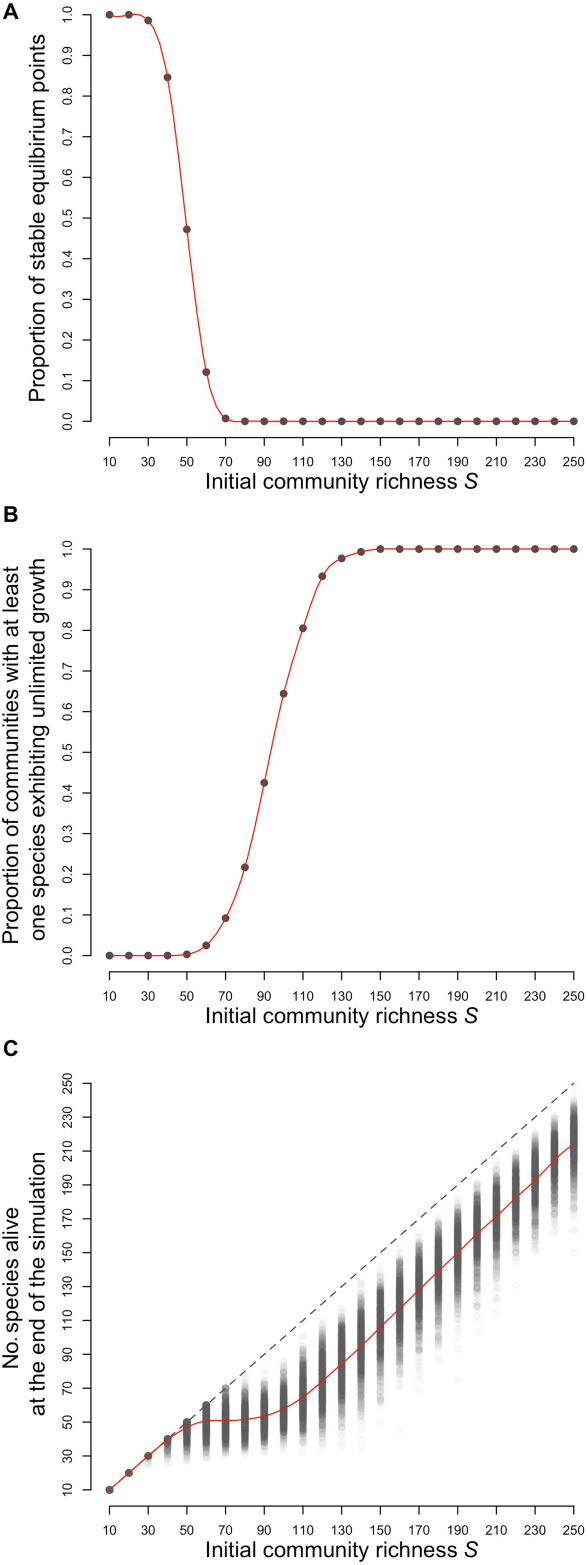
Effect of initial community richness on the outcome of the generalized Lotka–Volterra dynamics. Panel (A) represents the proportion of communities for which the equilibrium point is locally stable, that is, for which a small perturbation in the equilibrium abundance results in dynamics converging back to this equilibrium. Panel (B) shows the proportion of communities in which at least one species displays unlimited growth. Panel (C) shows the number of species alive at the end of the simulations. In panels (B and C), the simulations are stopped either if the system converges to a new equilibrium point or if at least one species reaches an abundance of 1000. The parameters for sampling the interaction matrices are *c* = 0.7 and σ = 0.2.

**FIGURE 3:**
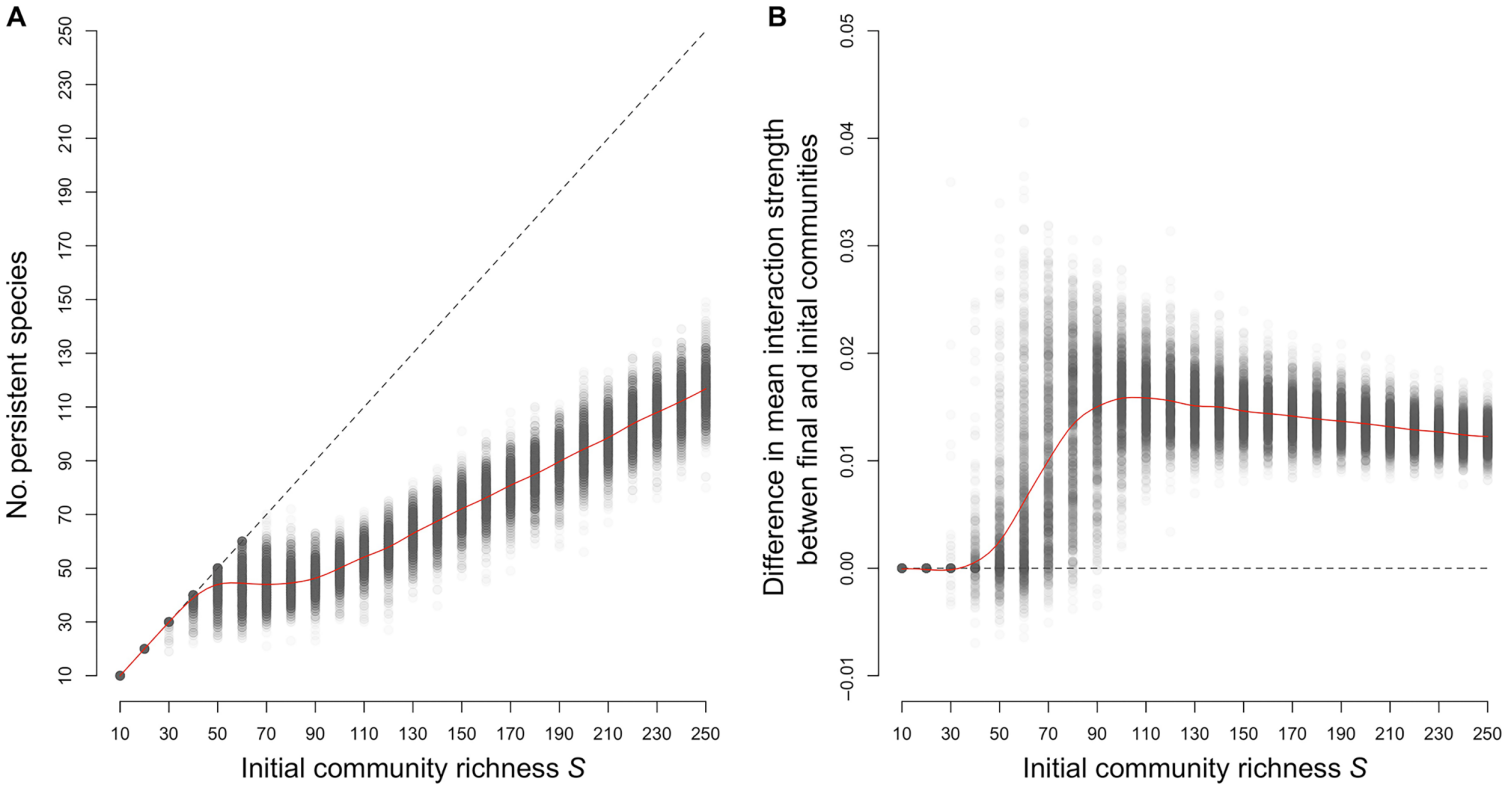
Effect of the initial community richness on the outcome of the modified generalized Lotka–Volterra dynamics. Panel (A) shows, as expected, that the number of persistent species is lower than the number of initial species once the local stability of the initial equilibrium point has been lost. However, most importantly, the number of persistent species still increases when increasing the initial community richness. Panel (B) shows that, in the final community made up of the persistent species, the per capita interaction strength is, on average, greater than that in the initial community, demonstrating that positive interactions are ecologically selected. The parameters for sampling the interaction matrix are the same as those in Figure 2.

**FIGURE 4:**
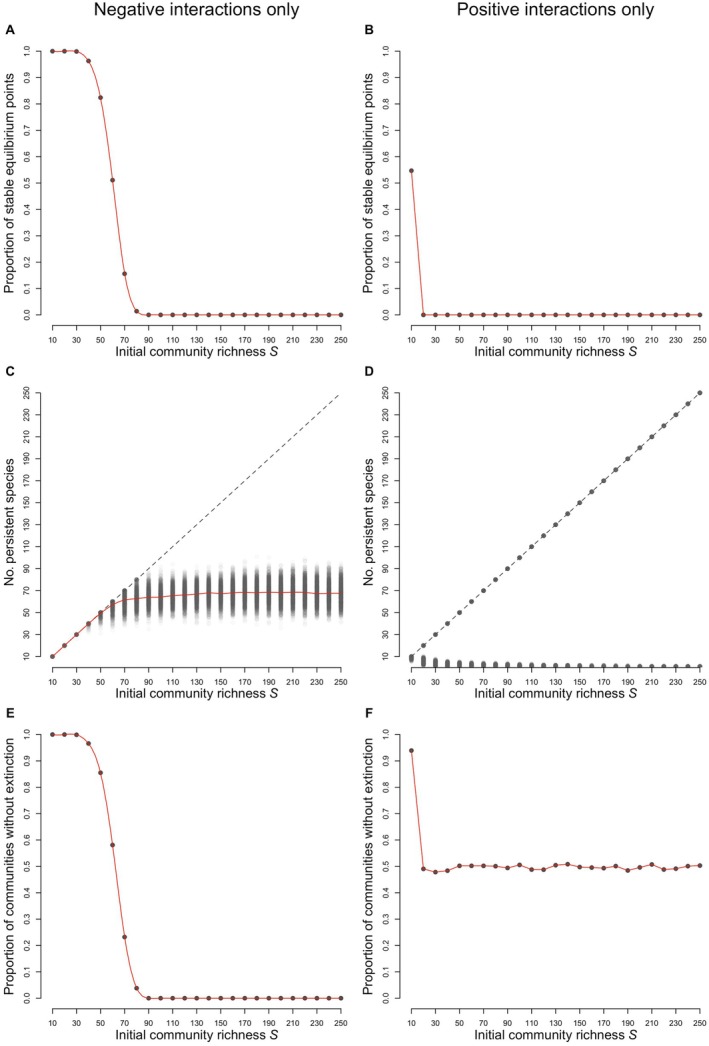
Effects on the dynamics outcome when the interspecific interactions are either all negative (competition and amensalism; panels A, C, E) or all positive (mutualism and commensalism; panels B, D, F). All intraspecific interactions are negative, and the parameters for sampling the interaction matrix are the same as those in Figure 2. Local stability is lost very rapidly (panels A and B). Panel D displays contrasting outcomes for the initially unstable equilibria with positive interactions: either the system converges to a new equilibrium without extinction (points on the main diagonal), or almost all species become extinct (points close to the abscissa). The probability of either of the outcomes occurring is approximately 0.5 (panel F).

Our approach attempts to remain as close as possible to the original May approach with the crucial addition that, with numerical simulations of the GLV model, we are able to follow the time course of population abundances, particularly in the cases of unstable equilibria. The main results are presented in Figures 2, 3, 4.

In Figure 2, we represent the proportion of communities with a nontrivial stable equilibrium point (Figure 2A), the proportion of communities with at least one species exploding to 1000 (Figure 2B), and the number of species alive at the end of the simulation (Figure 2C), as functions of the initial community richness *S*. Figure 2A confirms the original May results. As *S* increases, the probability that the equilibrium point is stable is 1 for lower richness (S<50 in our example), but it declines very fast for higher richness, even declining to zero for sufficiently large *S*. Indeed, complexity begets instability. However, do unstable systems become extinct? In Figure 2B, we can see that this is clearly not the case. This panel shows that, in rich unstable communities, at least one species exhibits unlimited growth. Moreover, Figure 2C shows that species‐rich communities can be obtained: this panel shows the number of species still alive at the end of the simulation. We observe that a number of species go extinct (because the red curve is lower than the dashed diagonal). However, after a transition, the number of species remaining alive increases linearly with increasing *S*, meaning that the proportion of lost species decreases. Very large viable communities can be built, and the greater the initial richness is, the greater the viable community richness.

In the above simulations, populations can grow with no limit: any population reaching the value of 1000 is considered on its way to infinity. For more realistic modeling, the GLV model is modified with an attenuation factor that sets $N_{\max}$ as the asymptotic limit that no population can exceed (see *Methods*). In Figure 3, we represent the number of live species at equilibrium (i.e., the number of persistent species) as a function of the initial richness *S*. We also calculate the change in the mean interaction strength, that is, the difference between the mean interaction strength among the species alive at equilibrium and the mean interaction strength among all initial species. A deviation from zero indicates ecological selection of interactions, which can be either positive (tendency to select positive interactions) or negative (tendency to select negative interactions).

Figure 3A shows that the modified GLV model does not alter qualitatively the main result of Figure 2C: starting from an initial community of richness *S*, a number of species are lost, but arbitrarily rich viable communities can still be built, which is shown by the fact that the average number of persisting species increases with no limit (red curve). Figure 3B shows that there is ecological selection of positive interactions: the mean interaction strength in the viable communities is greater than that in the initial pool.

The crucial role of the sign of mutual interactions is illustrated by Figure 4, in which we represent the proportion of communities for which no species becomes extinct. Species richness is still detrimental to stability, whether the interactions are all negative (Figure 4A) or all positive (Figure 4B). If they are all negative, persistent systems are readily obtained, but the number of persistent species cannot exceed a certain number (approximately 60–70 in the example of Figure 4C). If the interactions are all positive, contrasting outcomes are obtained at random (Figure 4D): either the system converges to a new equilibrium with all species alive (points on the diagonal) or almost all species go extinct (lower points, close to the *x*‐axis). These two outcomes occur with an approximate 50–50 probability (Figure 4F).

Finally, we checked with additional simulations that the patterns illustrated by Figures 2, 3, 4 are not qualitatively sensitive to connectance *c* and/or to interaction strength variability σ (see Appendix S1: Figures S1–S11). The effects are quantitative only, with no remarkably qualitatively new pattern.

As previously mentioned, none of the numerous simulations had to be stopped with the time limit criterion. In the unmodified GLV model, in all simulations, each population either reached a nonzero equilibrium, went extinct, or exploded. In the modified GLV model, all populations converged to an equilibrium (either zero or nonzero). This means that never‐ending trajectories like cyclic or chaotic regimes never occurred. We observed that alternative steady states do exist: when varying the perturbation only, one same system, with given parameters, can reach different equilibrium points, with different species surviving (see Appendix S1: Figure S12).

## DISCUSSION

The well‐known theoretical studies by R.M. May and followers seemed to contradict the fact that many complex viable ecosystems exist in nature. Our approach proposes a simple reconciliation. We argue that locally unstable systems must be considered viable when they escape some local equilibrium and grow to some other state (possibly losing a number of species but not all of them). Very species‐rich, complex viable systems can readily be built. Richness and/or connectance are not obstacles to system viability; on the contrary: the richer the species pool is, and the more complex interspecific interactions are, the more easily robust, highly efficient communities can be formed. These properties cannot be observed when using local stability as a proxy of viability (Allesina & Tang, 2012; Hatton et al., 2024; May, 1972, 1973; Mougi & Kondoh, 2012, 2014).

This is entirely due to the effect of positive interactions, not only in mutualistic and commensalistic situations, but also in antagonistic situations. In their absence, that is, in purely competitive systems, Figure 4C shows that species richness cannot exceed a certain limit. This limit depends on the connectance *c* and on the interaction strength variability σ (see Appendix S1: Figure S8): the higher σ and/or the higher *c*, the higher the interaction coefficients and the lower the numbers of coexisting species. In the language of niche theory, species packing limits the number of competing species that can coexist (MacArthur & Wilson, 1967). This result fully echoes the limiting similarity theory of competing species developed by MacArthur and Levins (1967) and its extension to species‐rich competition communities by Vandermeer (1970). These authors demonstrated theoretically that there is a maximum number of species that can coexist and that this number is a function of the mean interaction strength, which is in line with our results displayed in Figure 4C and in Appendix S1: Figure S8.

When only positive interactions are allowed, that is, in purely mutualistic systems, it is well known that this is a highly destabilizing situation (Allesina & Tang, 2012; Goh, 1979). In approximately half of the cases, all populations go extinct, as shown in Figure 4D. However, the same figure shows that, in the other half of the cases, all species survive and grow to their ceiling populations. Thus, although mutualism is locally destabilizing, it is a factor that helps persistence, not the opposite.

Positive interactions create ecological niches and increase the number of species that can coexist. This has recently been theoretically supported by Koffel et al. (2021) by extending the classical niche theory, which considers only competition for common resources, to include positive feedback loops that permit niche extension and, consequently, increase the number of coexisting species. Empirically, positive interactions have also been widely studied in plant communities (Callaway, 2007), such as nitrogen fixation, shade for seedlings, or pollen and seed dispersal by pollinators and other animals (Bascompte & Jordano, 2014). Our study shows the importance of positive interactions for maintaining and extending niches, allowing an increasing number of coexisting species.

Methodologically, the major innovation of our work is that, using numerical simulation of dynamic systems, we have been able to follow the time course of populations, thus escaping from the limited scope of local stability analyses in ecology. This answers the complaint of Allesina and Tang (2012) that, although natural systems generally operate far from a steady state, the theoretical study of large systems is still based on local stability for feasibility reasons. With the latter approach, the essential role of positive interactions is condemned to silence.

Choosing the GLV model to represent community dynamics can be questioned. Indeed, alternate models exist for pairwise interactions. Regarding pairwise competition, the original Lotka–Volterra model is generally accepted with no modification. Regarding pairwise mutualism, authors commonly introduce a saturation factor in order to avoid explosions (e.g., Mougi & Kondoh, 2012). Regarding pairwise predation, the Lotka–Volterra model of predator–prey interactions has been criticized, particularly by one of the present authors (Arditi & Ginzburg, 2012), because the model ignores predator saturation and predator interference. A large number of alternate predator–prey models have been proposed by many authors (e.g., Arditi & Ginzburg, 1989). However, it has proven impossible to generalize these alternate models to situations with more than one prey species and more than one predator species: all attempts failed to satisfy fundamental logical criteria (Arditi & Michalski, 1996). The GLV model remains irreplaceable in abstract theoretical studies such as the present article.

## AUTHOR CONTRIBUTIONS

*Concepts*: Roger Arditi, Rudolf P. Rohr, and Louis‐Félix Bersier. *Methods*: Rudolf P. Rohr and Roger Arditi. *Investigation*: Rudolf P. Rohr, Roger Arditi, and Louis‐Félix Bersier. *Figures*: Rudolf P. Rohr and Roger Arditi. *Writing—first draft*: Roger Arditi. *Writing—further versions*: Roger Arditi, Louis‐Félix Bersier, and Rudolf P. Rohr.

## CONFLICT OF INTEREST STATEMENT

The authors declare no conflicts of interest.

## Supporting information


Appendix S1:

## Data Availability

The R code (Rohr, 2025) for performing the simulations and for drawing the figures is available in Figshare at https://doi.org/10.6084/m9.figshare.28378022.v1.
