# Supplementary material for: Will a large complex model ecosystem be viable? The essential role of positive interactions
Source: Ecology. 2025 Mar 19;106(3):e70064. doi: 10.1002/ecy.70064 (PMC11920939; doi:10.1002/ecy.70064)
Supplement: Supplementary file 1 — Appendix S1: [file ECY-106-e70064-s001.pdf]

Ecology

Appendix S1

to the article

Will a large complex model ecosystem be viable? The essential role of positive interactions

by

Rudolf P. Rohr, Louis-Félix Bersier, Roger Arditi

In this appendix, the supplementary figures S1 to S11 are provided to demonstrate the effects of the parameters σ and c on the patterns illustrated by Figs. 2 to 4 of the main document. The figures show that the effects are quantitative only, with no remarkable qualitatively new pattern.

Figure S12 supports the statement in the last sentence of the Results section in the main document: the figure shows that alternative steady states exist and that they do not change our findings.

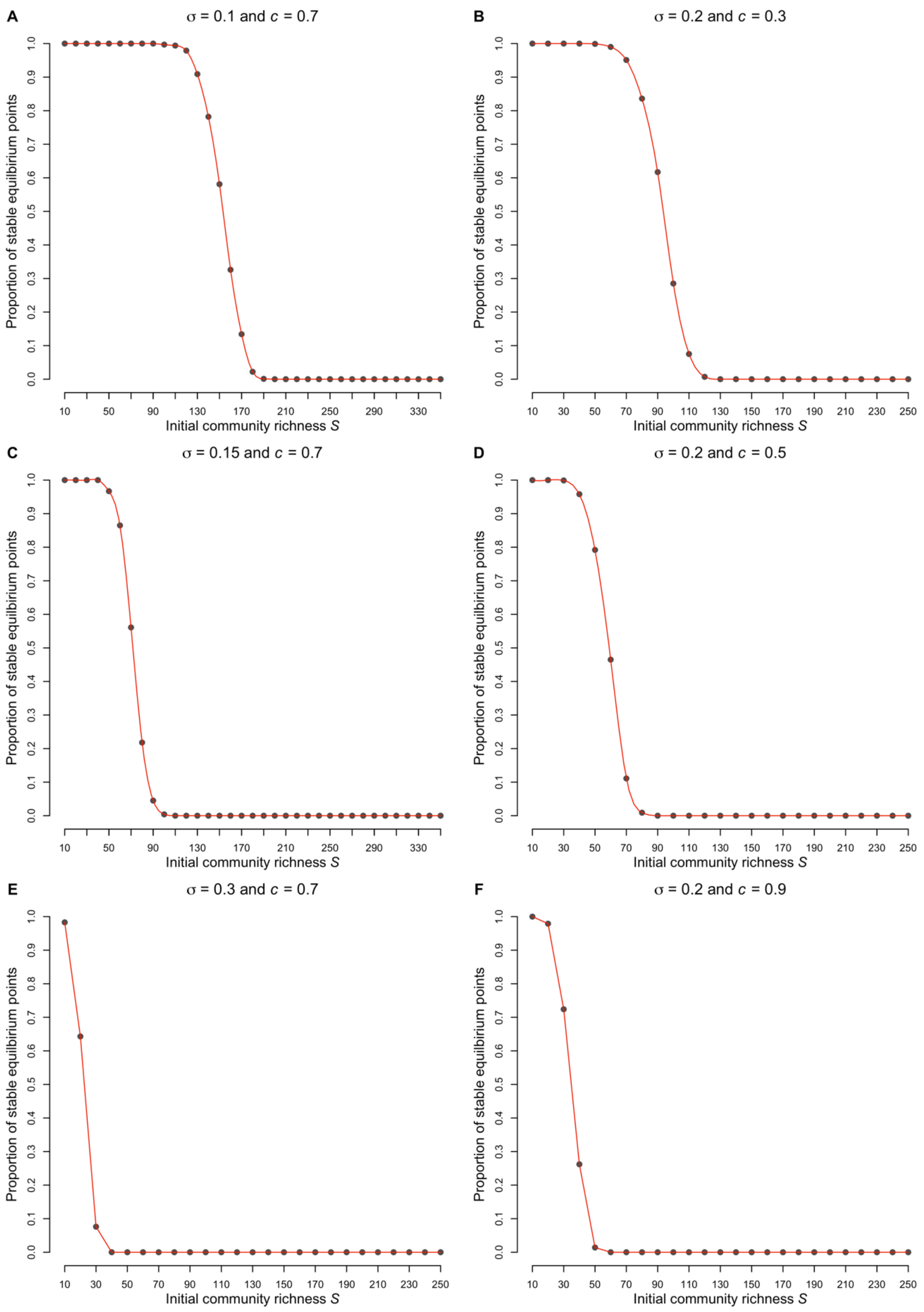

Figure S1. Analogous to Fig. 2A in the main text, with various values of σ and c.

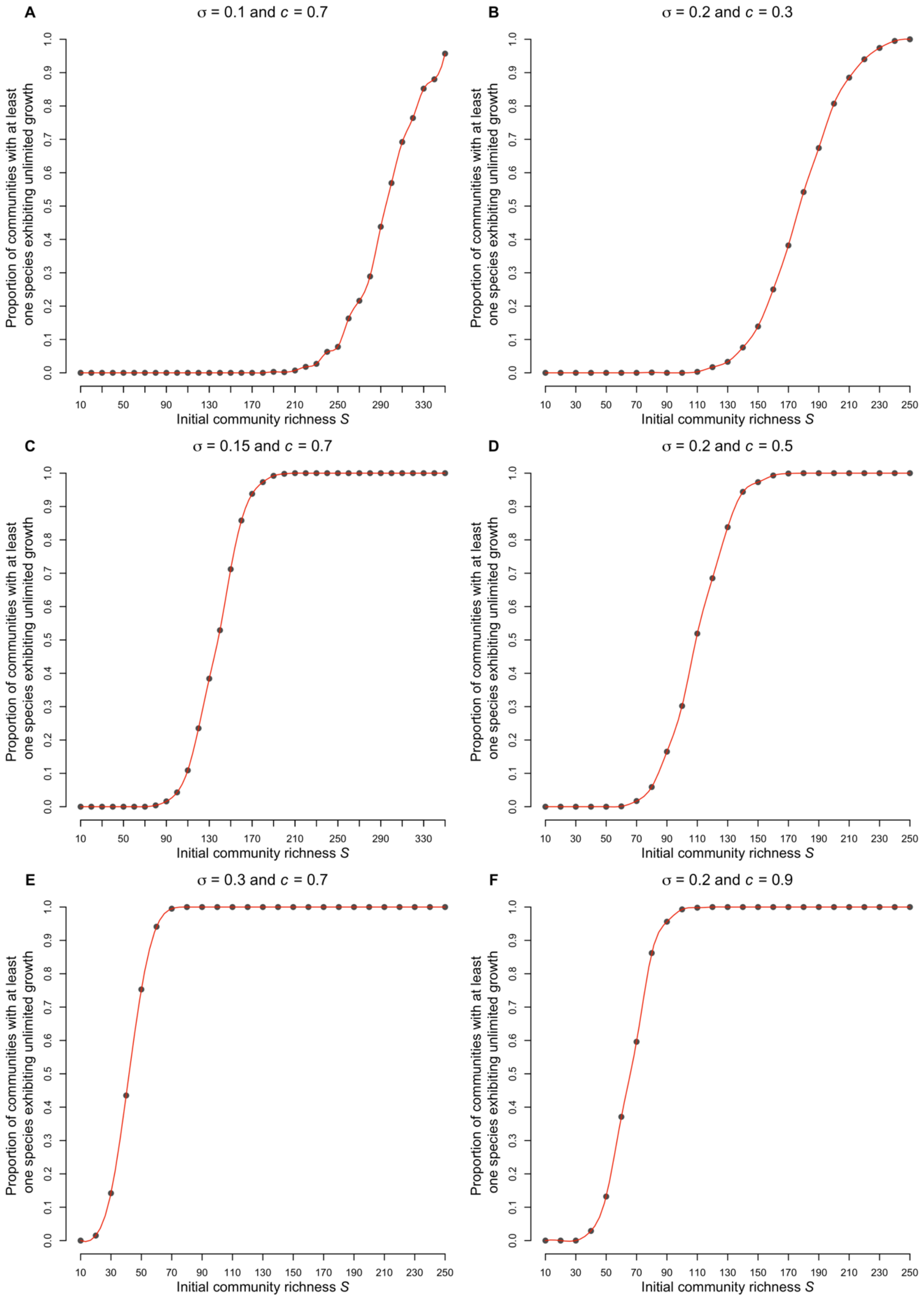

Figure S2. Analogous to Fig. 2B in the main text, with various values of σ and c.

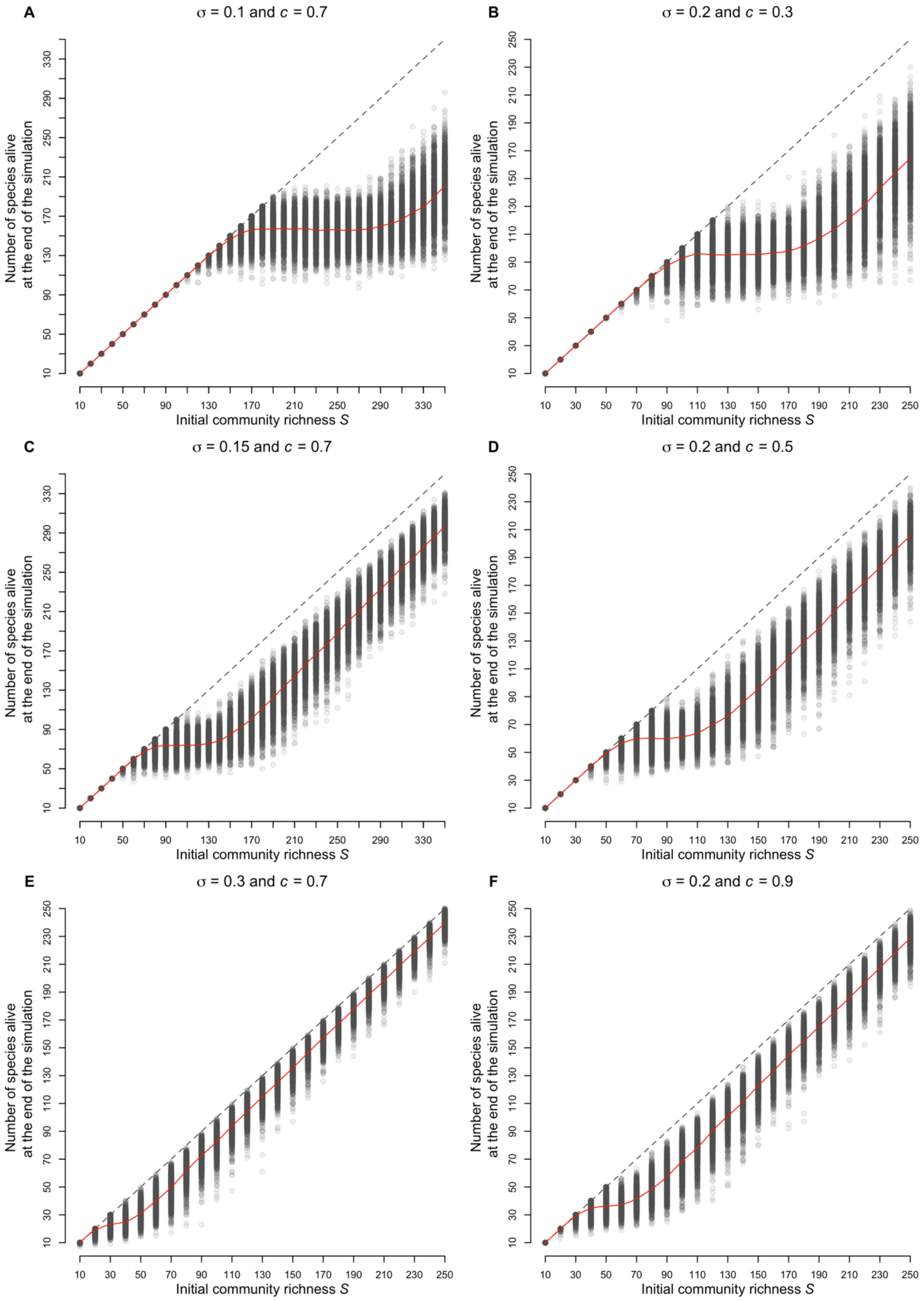

Figure S3. Analogous to Fig. 2C in the main text, with various values of σ and c.

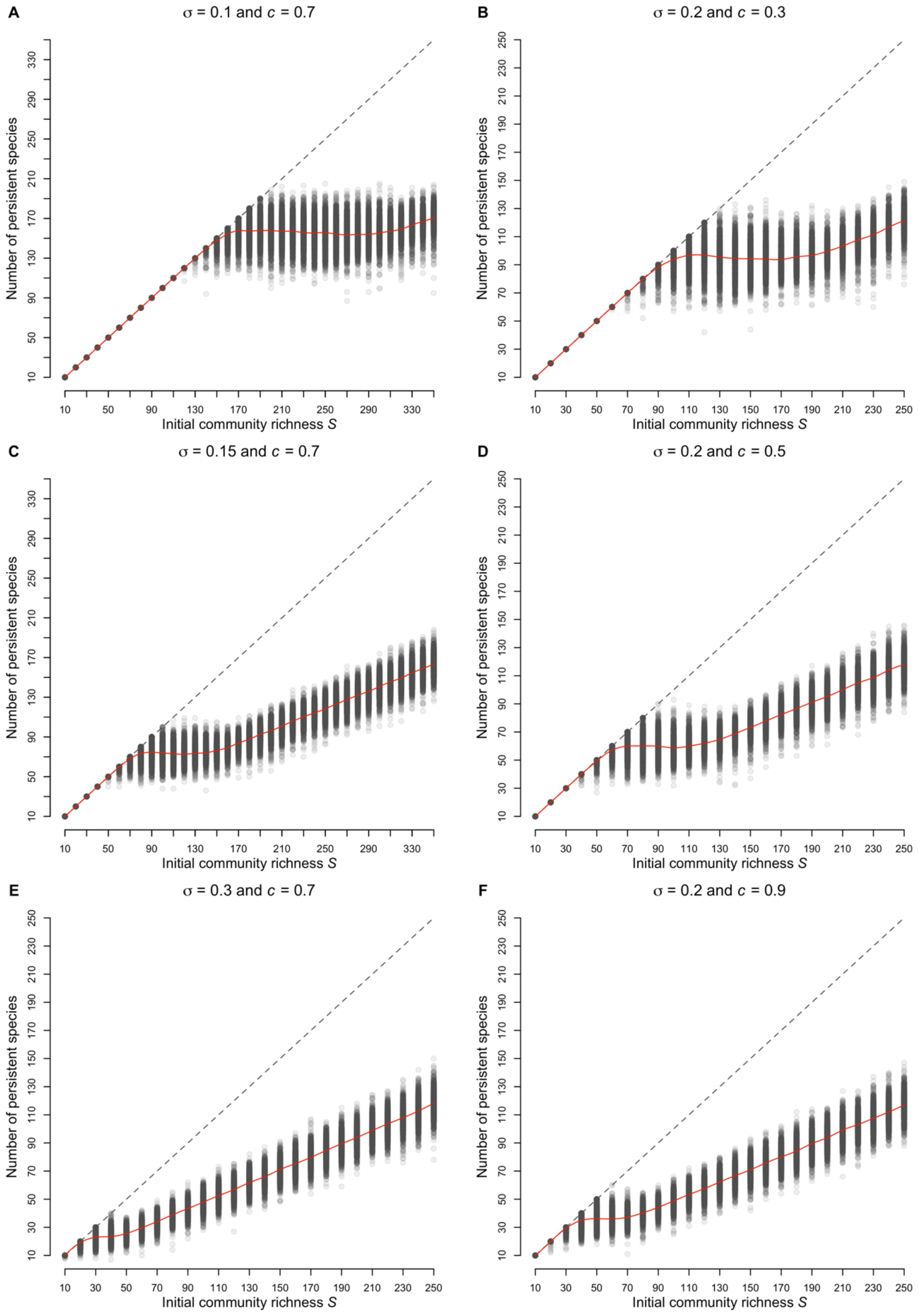

Figure S4. Analogous to Fig. 3A in the main text, with various values of σ and c.

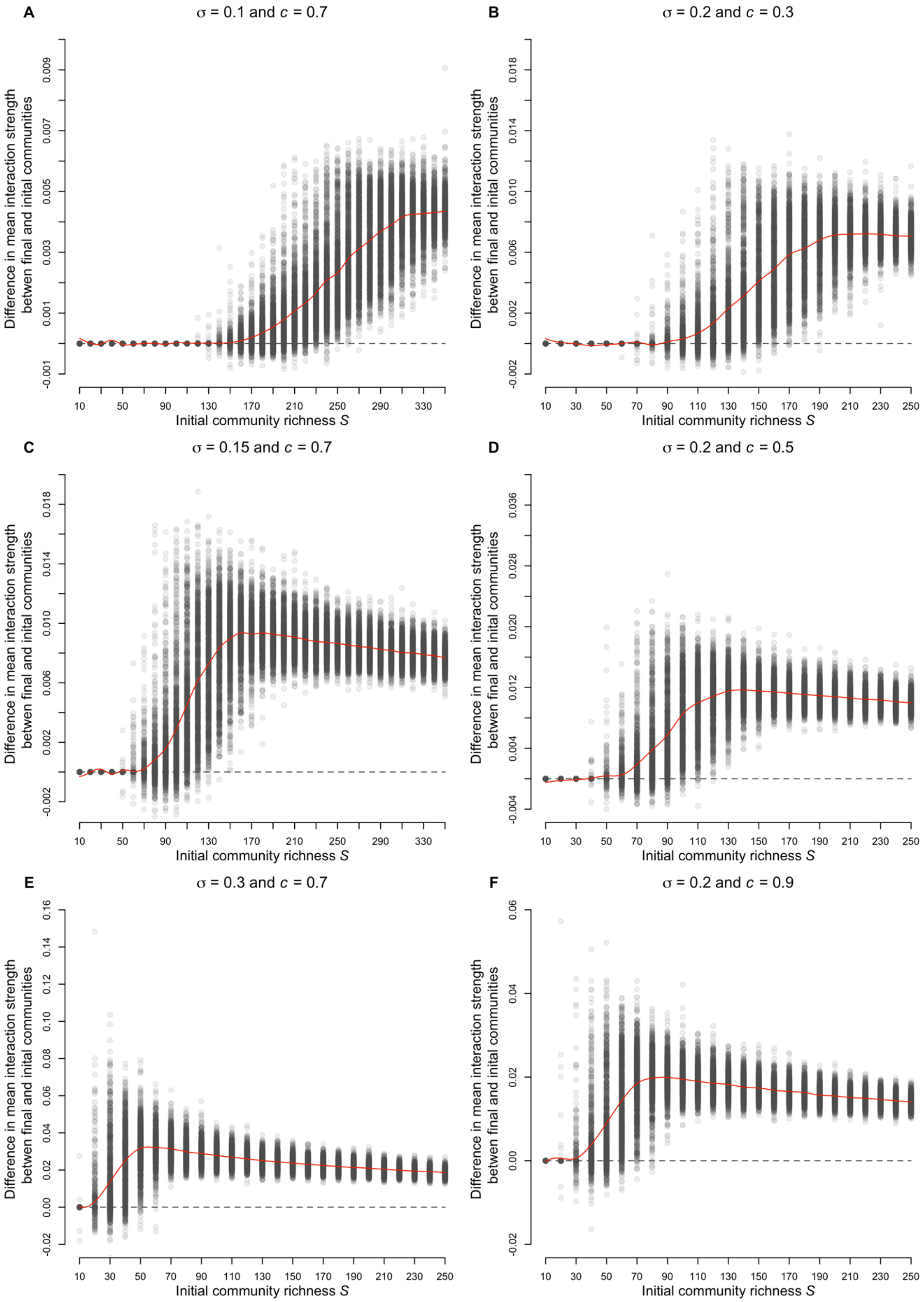

Figure S5. Analogous to Fig. 3B in the main text, with various values of σ and c.

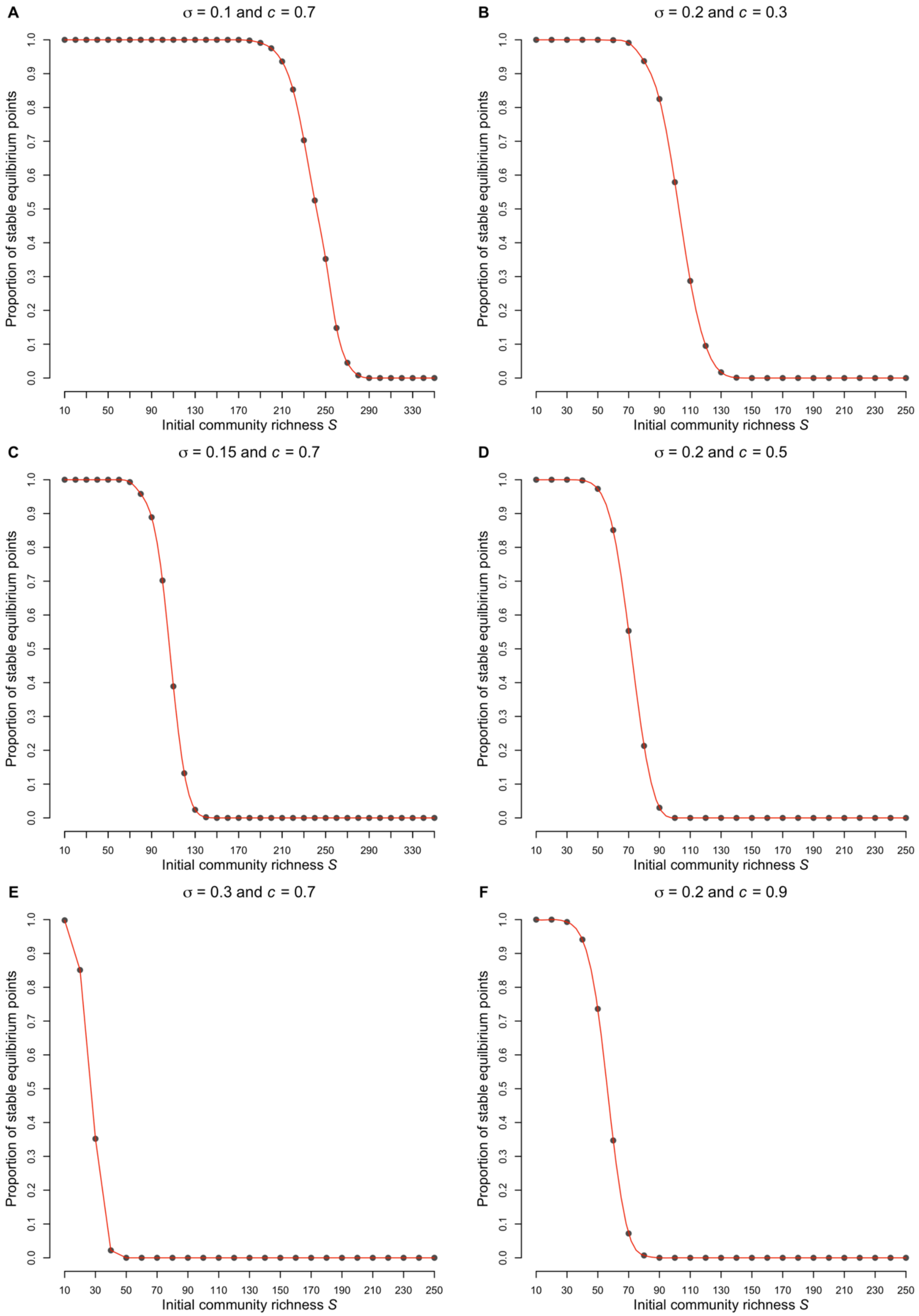

Figure S6. Analogous to Fig. 4A in the main text, with various values of σ and c.

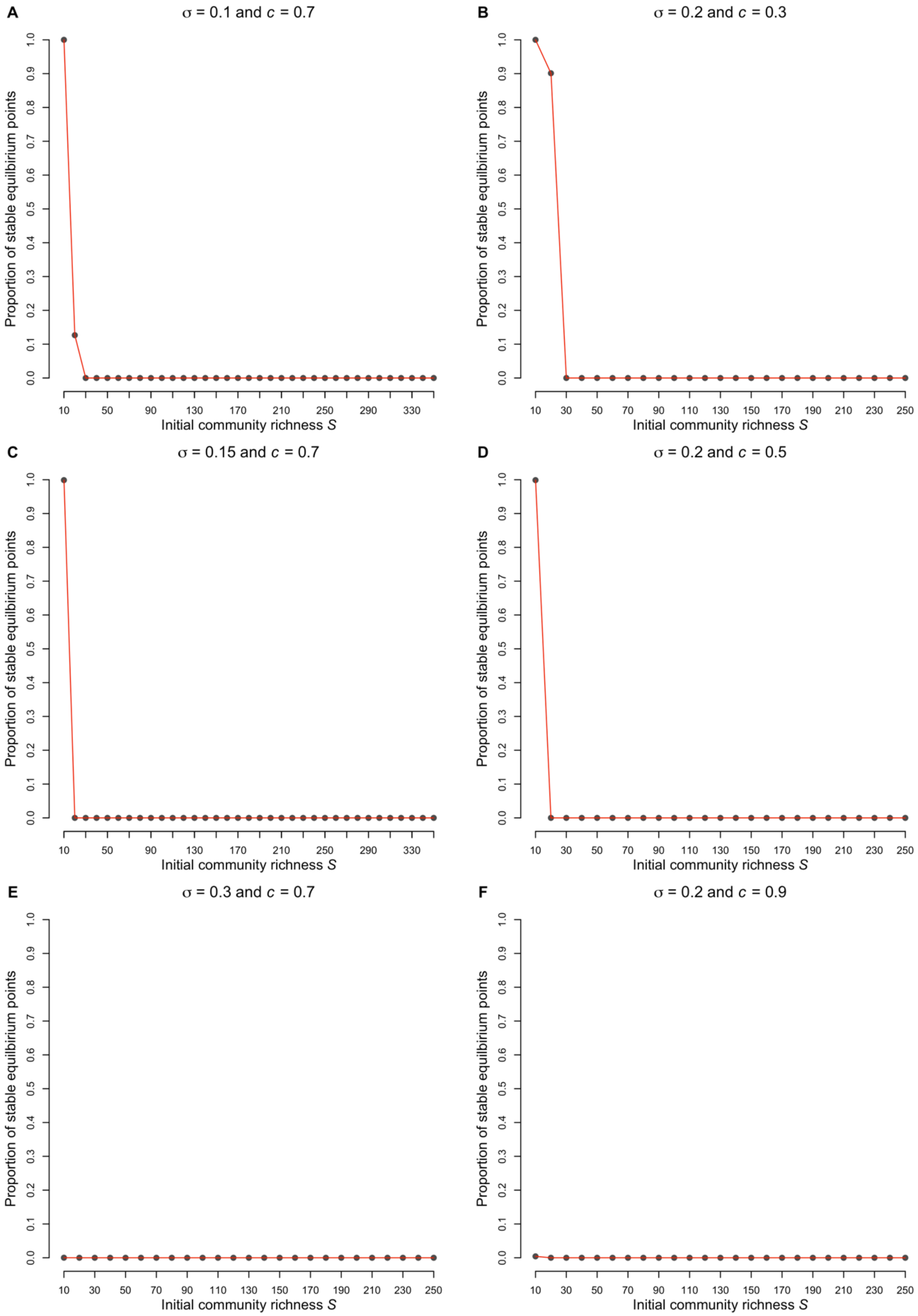

Figure S7. Analogous to Fig. 4B in the main text, with various values of σ and c.

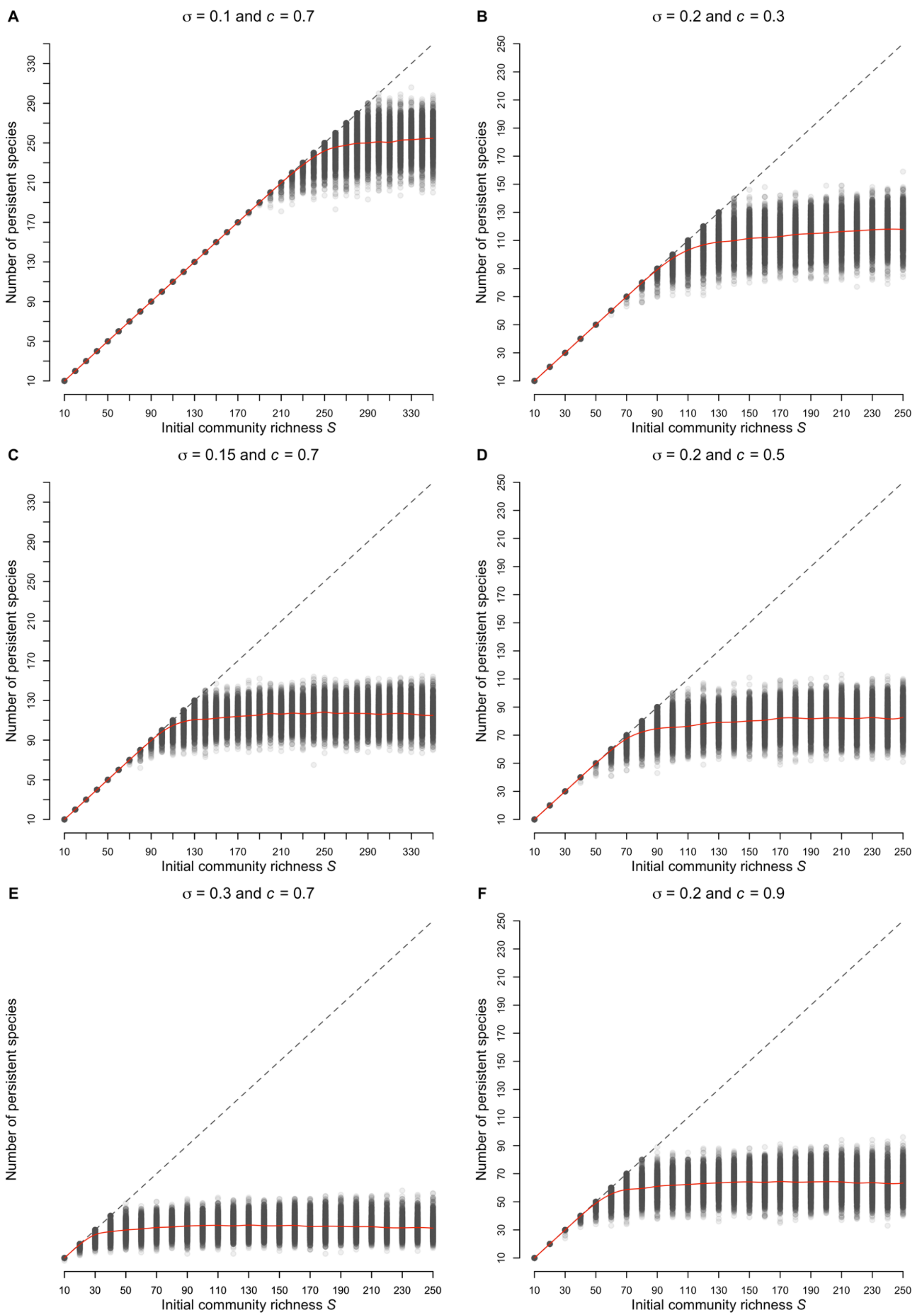

Figure S8. Analogous to Fig. 4C in the main text, with various values of σ and c.

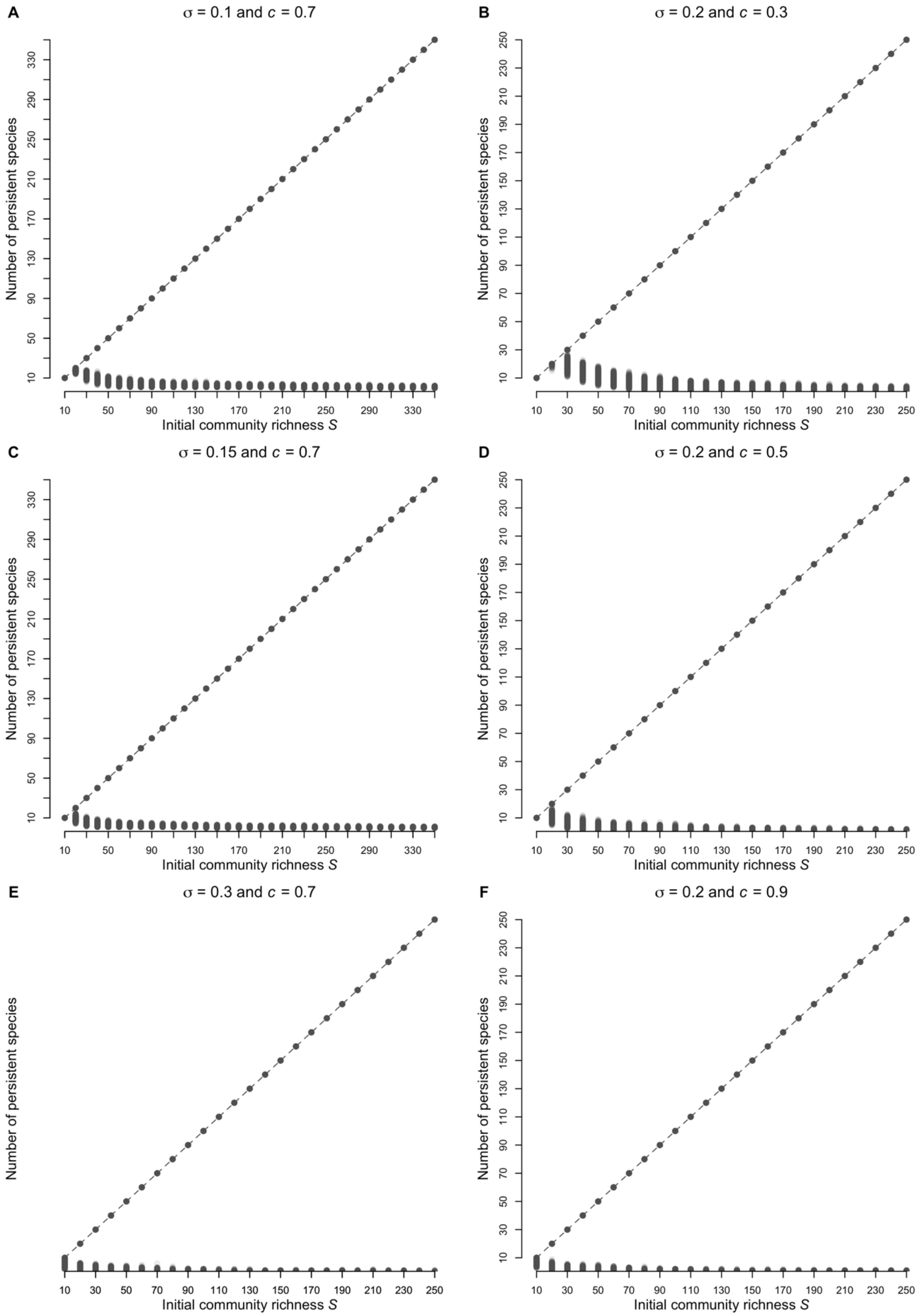

Figure S9. Analogous to Fig. 4D in the main text, with various values of σ and c.

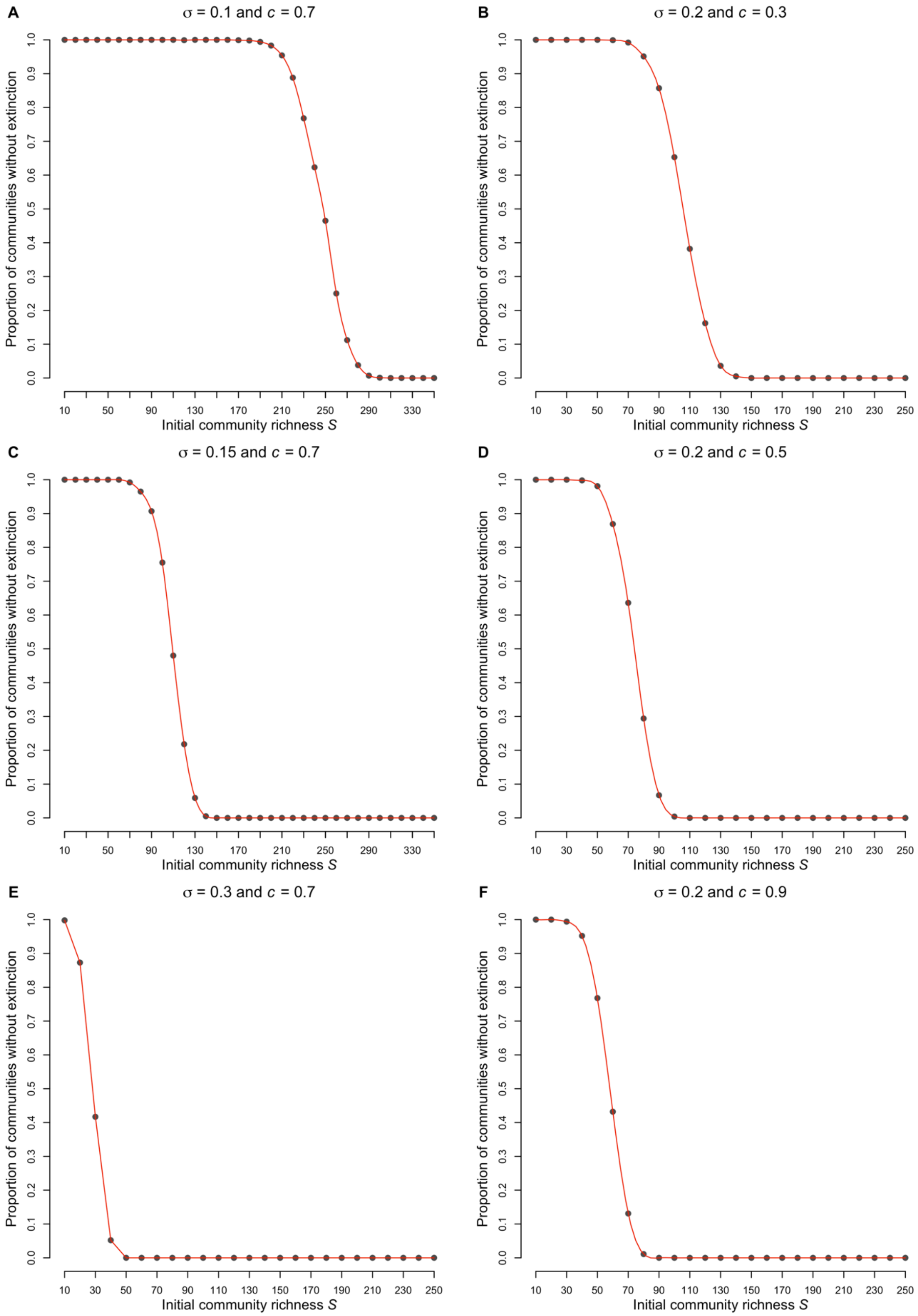

Figure S10. Analogous to Fig. 4E in the main text, with various values of σ and c.

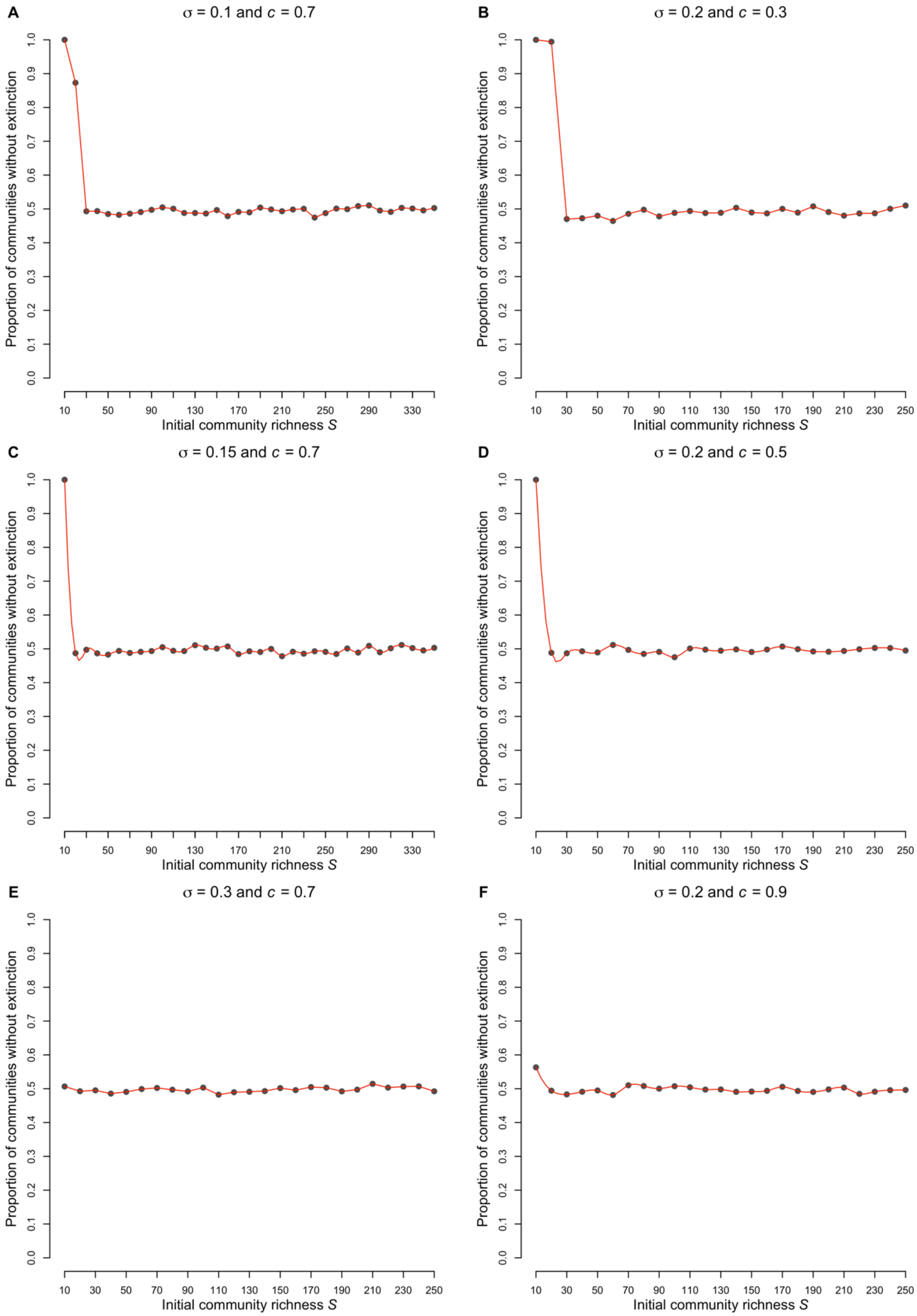

Figure S11. Analogous to Fig. 4F of the main text, with various values of σ and c.

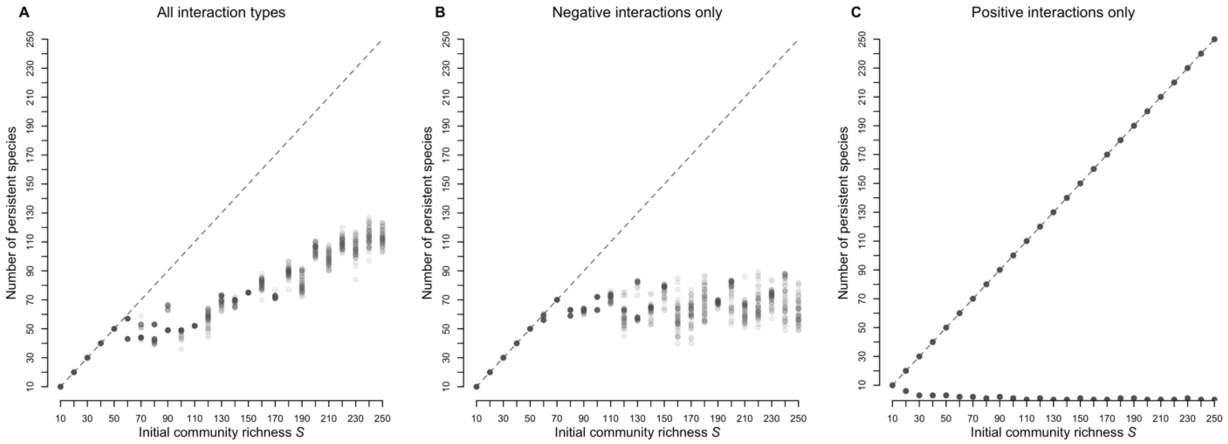

Figure S12. This figure shows that alternative steady states exist and that they do not change our conclusions. The parameters for sampling the interaction matrices are c=0.7 and σ=0.2. For any given value of S, the various vertically aligned points result from different perturbations of the same initial equilibrium and the same interaction matrix. Since the points are spread vertically, this means that the various simulations reach different final states, with different species surviving.
